# Comparing development and regeneration in the submandibular gland highlights distinct mechanisms

**DOI:** 10.1111/joa.13387

**Published:** 2021-01-16

**Authors:** Lemonia Chatzeli, Tathyane H. N. Teshima, Mohammad K. Hajihosseini, Marcia Gaete, Gordon B. Proctor, Abigail S. Tucker

**Affiliations:** ^1^ Centre for Craniofacial and Regenerative Biology King's College London London UK; ^2^ Department of Oral Medicine UCL Eastman Dental Institute London UK; ^3^ School of Biological Sciences University of East Anglia Norwich UK; ^4^ Department of Anatomy Faculty of Medicine Pontificia Universidad Católica de Chile Santiago Chile; ^5^ Centre for Host‐Microbiome Interactions King's College of London London UK

**Keywords:** adult, duct, Fgf10, ligation, remodelling, repair, Sox9

## Abstract

A common question in organ regeneration is the extent to which regeneration recapitulates embryonic development. To investigate this concept, we compared the expression of two highly interlinked and essential genes for salivary gland development, *Sox9* and *Fgf10*, during submandibular gland development, homeostasis and regeneration. Salivary gland duct ligation/deligation model was used as a regenerative model. Fgf10 and Sox9 expression changed during regeneration compared to homeostasis, suggesting that these key developmental genes play important roles during regeneration, however, significantly both displayed different patterns of expression in the regenerating gland compared to the developing gland. Regenerating glands, which during homeostasis had very few weakly expressing Sox9‐positive cells in the striated/granular ducts, displayed elevated expression of Sox9 within these ducts. This pattern is in contrast to embryonic development, where Sox9 expression was absent in the proximally developing ducts. However, similar to the elevated expression at the distal tip of the epithelium in developing salivary glands, regenerating glands displayed elevated expression in a subpopulation of acinar cells, which during homeostasis expressed Sox9 at lower levels. A shift in expression of Fgf10 was observed from a widespread mesenchymal pattern during organogenesis to a more limited and predominantly epithelial pattern during homeostasis in the adult. This restricted expression in epithelial cells was maintained during regeneration, with no clear upregulation in the surrounding mesenchyme, as might be expected if regeneration recapitulated development. As both Fgf10 and Sox9 were upregulated in proximal ducts during regeneration, this suggests that the positive regulation of Sox9 by Fgf10, essential during development, is partially reawakened during regeneration using this model. Together these data suggest that developmentally important genes play a key role in salivary gland regeneration but do not precisely mimic the roles observed during development.

## Graphical abstract

A common question in organ regeneration is the extent to which regeneration recapitulates embryonic development. To investigate this concept, we compared the expression of two highly interlinked and essential genes for salivary gland development, *Sox9* and *Fgf10*, during submandibular gland development, homeostasis and regeneration. Our data suggest that developmentally important genes play a key role in salivary gland regeneration but do not precisely mimic the roles observed during development.

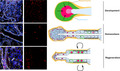

## 1 INTRODUCTION

Regeneration of organs is often proposed to mimic development with re‐activation of developmental signalling pathways and master regulators during repair (Cotroneo et al., 2010; Patel & Hoffman, 2014; Villanueva et al., 2006; Xu et al., 2008). To develop therapeutic strategies for organ regeneration, studies have therefore focused on understanding how organs are formed normally during embryogenesis. However, a common question that emerges is the extent that regeneration recapitulates embryonic development. Evidence from branching organs, including the pancreas and kidneys, indicates a re‐activation of some developmental pathways and transcription factors during regeneration, linking these two processes (Villanueva et al., 2006; Xu et al., 2008).

In the developing pancreas both the endocrine and exocrine cell lineages derive from a common precursor (Akiyama et al., 2005), while during homeostasis, the endocrine and exocrine cells are derived by self‐duplication (Desai et al., 2007; Dor et al., 2004). Pancreatic duct ligation model of injury, although still debatable (Yu et al., 2016), activates a population of adult endocrine progenitors located in the ducts. These adult progenitors re‐express the transcription factor Neurogenin 3 (Ngn3), the earliest marker of islet precursors during development (Xu et al., 2008). In the case of exocrine cell regeneration, the remaining acinar cells dedifferentiate to an embryonic‐like progenitor state during acute injury and activate developmental pathways (Gu et al., 2002; Jensen et al., 2005).

The kidney is another example where multiple developmental genes have been shown to be expressed during kidney regeneration after acute kidney injury (Bonventre, 2003; Devarajan et al., 2003; Little & Kairath, 2017; Villanueva et al., 2006). Among these upregulated genes is the transcription factor Sex Determining Region Y‐Box 9 (Sox9), which is involved in branching morphogenesis during kidney development (Kumar et al., 2015; Reginensi et al., 2011). In the adult kidney, Sox9 is expressed at low levels in almost all of the tubule segments and is upregulated upon acute kidney injury (Kang et al., 2016; Kumar et al., 2015). The Sox9 expressing population is the main source of proliferating cells during the regeneration process and they are required for normal repair (Kang et al., 2016).

Given these similarities and differences between development and regeneration in these different branching organs, we have turned to salivary glands to investigate the links between development and repair. Salivary glands have a slow turnover ranging from 50 to 125 days (Aure et al., 2015), however, they have a remarkable ability to regenerate after certain types of tissue injury (Carpenter et al., 2007). Three pairs of major salivary glands exist in mammals; the parotid, the submandibular and the sublingual gland. These glands vary in the type of saliva produced, their branching pattern and therefore their function. They all contain the saliva producing acinar cells surrounded by myoepithelial cells and a well‐developed ductal tree formed by intercalated, granular/striated and excretory ducts that drain saliva into the oral cavity (Tucker, 2007). Understanding the mechanism of salivary gland regeneration is important due to the therapeutic potential in enhancing organ regeneration. Salivary glands are often damaged by irradiation treatment in head and neck cancer patients (10% of malignant tumours) (Vissink et al., 2003), by autoimmune disorders in patients with Sjögren's syndrome (Young et al., 2001) and by ageing, affecting around 27% of the elderly (Gupta et al., 2006), and in these cases the glands do not naturally regenerate. Salivary gland dysfunction often leads to xerostomia, a feeling of dry mouth, which impairs speech, digestion and oral health (Plemons et al., 2014). Despite its significance, the current therapeutic options, such as the use of salivary stimulants, are inefficient as they only provide temporary relief from symptoms or rely on the pre‐existence of some functional tissue (Plemons et al., 2014). Thus, there is a lot of interest in understanding what happens in the cases where salivary glands are able to regenerate after injury.

Salivary gland duct ligation is one of the most commonly used methods to study regeneration in rodents (Bozorgi et al., 2014; Carpenter et al., 2007). This method involves the obstruction of the main excretory duct which leads to saliva retention, increase tissue pressure and cell lysis (Cotroneo et al., 2010). As a result, the injured tissue undergoes extensive acinar cell loss, duct dilation and extensive fibrosis and inflammation (Cotroneo et al., 2010). Once the duct obstruction is released, acinar cells are produced by a process that involves self‐duplication of the remaining acinar cells (Aure et al., 2015). Potential activation of an adult stem/progenitor cells found in the ducts has been suggested (Pringle et al., 2013), but this only appears to occur in extreme cases of damage (Weng et al., 2018). Based on anatomical observations and activation of certain developmental pathways, it has been suggested that the process of salivary gland regeneration mimics embryonic development (Cotroneo et al., 2010; Patel & Hoffman, 2014). Anatomical observations include the appearance of embryonic‐like branched structures ending in acinar cells during regeneration, suggesting a recapitulation of the branching process during repair (Cotroneo et al., 2010). Signalling pathways upregulated include Wnt signalling that is reactivated in the ductal cells of regenerating glands (Hai et al., 2010), similar to embryonic glands during differentiation (Patel et al., 2011). Signals provided by the parasympathetic nervous system that innervates the glands have also been shown to be necessary during embryonic salivary gland branching morphogenesis and regeneration (Knox et al., 2013; Proctor & Carpenter, 2007). In addition, pharmacological activation of Fibroblast Growth Factor (Fgf) and Ectodysplasin A (Eda) signalling, two pathways required for epithelial development of salivary glands (Entesarian et al., 2005; Jaskoll et al., 2005; May et al., 2015; Teshima et al., 2016; Wells et al., 2010), partially restore the function and the anatomy of irradiated salivary glands (Hill et al., 2014; Lombaert et al., 2008).

Salivary glands, like kidneys, pancreas and lung, are formed through branching morphogenesis, a process which is driven by the distal part of the epithelium (tips of the gland) (Chatzeli et al., 2017). Sox9 is a transcription factor expressed in the initial epithelial progenitors of the gland placode and later, during branching morphogenesis, in the distal epithelium (Chatzeli et al., 2017). In the absence of *Sox9*, branching initiation is inhibited due to the depletion of the distal epithelial progenitors (Chatzeli et al., 2017). Sox9 has also been shown to be one of two transcription factors that can reprogramme mouse embryonic stem cells to a salivary gland fate (Tanaka et al., 2018). *Fgf10* is expressed in the neural crest‐derived mesenchyme at the initiation stage and throughout branching morphogenesis and is required both for the initial stages of bud formation and for branching morphogenesis (Jaskoll et al., 2005; Wells et al., 2013). Fgf10 signals to the epithelium through the receptor FgfR2 (Jaskoll et al., 2002; Ohuchi et al., 2000; Steinberg et al., 2005). *Fgf10*‐null or *FgfR2*‐null salivary glands develop a rudimentary prebud with no further growth while pharmacological inhibition of Fgf signalling inhibits branching morphogenesis (Jaskoll et al., 2005; Ohuchi et al., 2000). A similar phenotype is observed after conditional knockout of *Fgf10* in the neural crest‐derived mesenchyme, confirming the importance of Fgf10 produced from this tissue during development (Teshima et al., 2016). One of the roles of Fgf10 during development is to maintain Sox9 expression in epithelial progenitors (Chatzeli et al., 2017), thus these pathways are closely interlinked during early development.

Given the importance of *Fgf10* and *Sox9* in salivary gland development, we have examined the pattern of expression of Fgf10 and Sox9 during salivary gland regeneration and homeostasis in the adult and compared this to the pattern during development.

## 2 METHODS

### 2.1 Mouse lines

Wild‐type mice were of CD1 strain. *Fgf10^nlacZ^* ^/+^ and *Wnt1^cre^* mice have been previously described (Danielian et al., 1998; Kelly et al., 2001). The *tdTomato* mouse (*Gt(ROSA)26Sor^tm14(CAG−tdTomato)Hze^* JAX Laboratories) was used as a reporter line crossed to *Wnt1^cre^* (Madisen et al., 2010). Due to sexual dimorphism of murine salivary glands, female mice only were selected for the surgical experiments. In contrast, male and female adult *Fgf10^nlacZ^* ^/+^ mice were analysed. All procedures and culling methods were compliant with UK Home Office regulations and with the approval of the King's College London Biological Safety committee. Mice were aged between 6 and 10 weeks for all analysis of adult expression.

### 2.2 Salivary gland duct ligation and deligation

Salivary gland ligation and deligation was performed in mice as previously described (Bozorgi et al., 2014). Female mice aged between 6 and 10 weeks were used for the surgery. Briefly, mice were anaesthetised with an intraperitoneal injection of Ketamine 75 mg/kg and Xylazine 15 mg/kg dissolved in PBS. A small incision was made at the neck region to expose the main excretory duct of the sublingual and the submandibular gland. Once the excretory duct was exposed, a titanium haemostatic micro clip (Mediplus) was applied using a clip applier (Mediplus). Ligation was performed only on the right side. The incision was then sutured with absorbable Vicryl‐coated sutures (Aston Pharma) and mice were left with the clip for 8 days. Mice were then either euthanised and their submandibular glands were removed or undergone a deligation procedure. For deligation, mice were re‐anaesthetised, an incision made and the clip removed. To evaluate the ligation efficiency, a bigger incision was made in some mice in order to expose the operated and contralateral unoperated salivary glands. Efficiency was based on the size of the gland and the formation of fibrotic tissue. Mice were then sutured and left to recover for 4 days. After that time they were euthanised and submandibular glands were removed for further analysis. Unoperated mice were used as a control.

### 2.3 X‐gal staining

Salivary glands from *Fgf10^nlacZ^* ^/+^ mice where dissected and fixed in 4% PFA for 20 min (Kelly et al., 2001). *Fgf10* ^+/+^ littermates were used as controls. Salivary glands were then washed for 5 min in PBS with 2 mM MgCl_2_ followed by another wash for 15 min. Glands were then incubated for 5 min in a solution containing 1 mM MgCl_2_, 0.2% NP‐40 and 0.02% deoxycholic acid diluted in PBS (Solution B). Glands were stained with Solution C made with 5 mM K_3_Fe(CN)_6_, 5 mM K_4_Fe(CN)_6_ and 1 mg/ml x‐gal diluted in Solution B. Staining was performed at 37°C for 4 h. Salivary glands were then washed in PBS 3 times for 5 min and processed for cryosectioning. Using the same protocol, cryosections on slides were additionally stained at 37°C for 4 days to increase the intensity of the signal, washed in PBS and re‐fixed. Sections were then dehydrated in EtOH series, counterstained with alcoholic Eosin and mounted with Neo‐Mount via Neo‐clear. Slides were photographed on a Nikon microscope.

### 2.4 Trichrome staining

Slides were dewaxed by incubating twice in Histoclear II (National Diagnostics) for 10 min, rehydrated in decreasing concentrations of IMS for 2 min and in dH_2_O for 2 min. For Alcian Blue staining slides were incubated in 1% Alcian Blue (Fluka) dissolved in 3% acetic acid (Analytical Reagents) pH2.5 for 10 min, rinsed briefly in dH_2_O and washed under running tap water for 10 min. For haematoxylin staining, slides were incubated in Ehrlich's haematoxylin (Solmedia) for 2 min, washed in running tap water for 10 min and rinsed in dH_2_O. For staining differentiation, slides were incubated in 2.5% phosphomolybdic acid (Fisher) for 10 min and rinsed in dH2O. Sirius Red staining was performed with 0.5% Sirius Red (BDH) in saturated picric acid (Fluka Biochemica) for 20 min and slides were rinsed twice in 0.5% acetic acid. After blotting dry, slides were washed 3 times in 100% IMS for 2 min each and for 5 min twice in histoclear. Slides were mounted with Neomount (Merck), coverslipped and left to dry at 40°C overnight. Alcian blue stains muccopolysaccharides blue while Sirius red in pircric acide stains collagens for visualisation of the connective tissue. Haematoxylin stains the nuclei.

### 2.5 Immunostaining and *in situ* hybridisation

Salivary gland tissue was embedded in paraffin as previously described (May et al., 2015). Immunofluorescence and *in situ* hybridisation was performed as previously described (Gaete et al., 2015). Primary antibodies and dilutions were used as follows: anti‐Fgf10 (Rabbit) 1:500 (ABN44, Millipore); anti‐Sox9 (Rabbit) 1:300 (AB5535, Millipore) and anti‐Mist1 (mouse) 1:100 (sc‐98771, Santa Cruz Biotechnology) which was used with the TSA kit for signal amplification (PerkinElmer), anti‐beta‐Galactosidase (chicken) 1:500 (Ab9361 Abcam), anti‐Ecadherin (mouse) 1:100 (ab76055 Abcam), anti‐RFP (rat) 1:200 (5F8 Chromotek) and anti‐PCNA (mouse) 1:100 (Thermofisher Scientific). All immunoresults were analysed on a confocal laser‐scanning microscope (TCS SP5, Leica), including negative and positive controls. Z stacks were created to confirm expression levels of Sox9 within the nucleus. Confocal images were processed using Image J and Photoshop software.

### 2.6 Cell quantification and statistical analysis

Cells were quantified manually using the cell counter plug in of Fiji/ImageJ (Schindelin et al., 2012). The mean of three different sections was calculated. Results were plotted and statistically analysed using Graph Pad Prism software. Statistical significance was calculated using unpaired t‐tests. Significance was taken as *p* < 0.05 (*), *p* < 0.01 (**) or *p* < 0.001 (***).

## 3 RESULTS

### 3.1 The expression of Sox9 in distal epithelium is maintained in adult submandibular glands but with altered levels of expression

**FIGURE 1:**
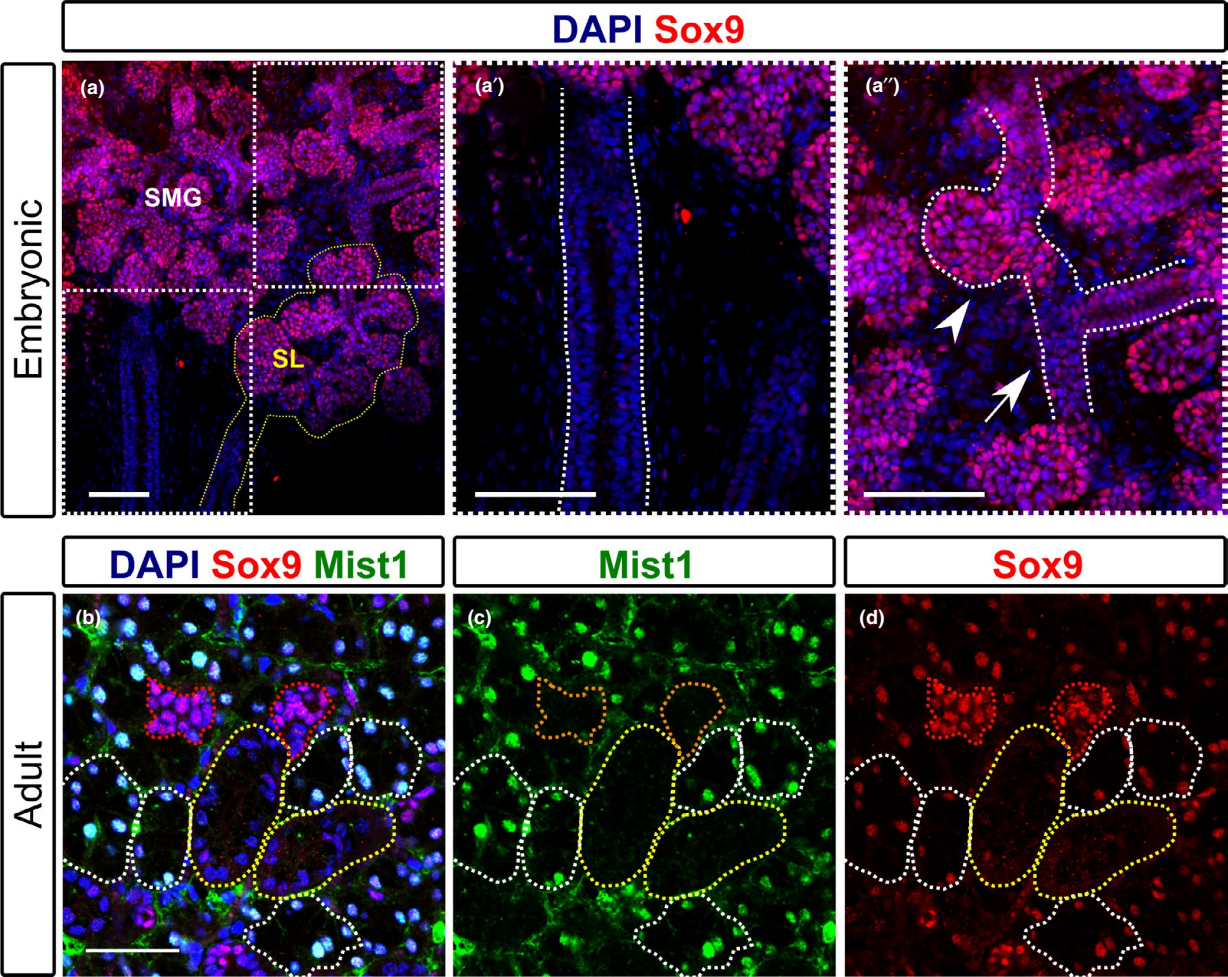
Sox9 expression in embryonic and adult submandibular glands. (a–a″) Immunofluorescence of Sox9 (pink) in the embryonic E15.5 submandibular glands. (a) The adjacent embryonic sublingual gland (outlined in dashed yellow), which displays a similar pattern of expression to the submandibular gland. Dotted boxes in (a) indicate the area magnified in a′ and a′. (a′) Submandibular gland main duct, outlined by dotted white lines. (a″) Arrow head indicates forming acini with high levels of Sox9 expression. Arrow indicated forming intercalated duct with lower levels of Sox9. (b–d) Immunofluorescence of Sox9 (pink) and Mist1 (green) in the adult submandibular gland during homeostasis (female mouse 6–10 weeks). DNA is shown in blue (DAPI). (b) DAPI, Sox9, Mist1. Red dotted lines outline small intercalated ducts. White dotted lines outline acini. Yellow dotted lines outline larger striated/granular ducts. (c) Nuclear Mist1 highlights acini cells. (d) Sox9 is highest in Mist1‐negative intercalated ducts. SMG, submandibular gland; SL; sublingual gland. Scale bars: 100 μm (a‐a″); 50 μm (b–d)

As a first approach to assess the role of Sox9 in the adult and embryonic salivary glands, the pattern of Sox9 expression in embryonic E15.5 and adult salivary glands was compared. At E15.5, a massive expansion of the epithelium through branching morphogenesis occurs along with the initiation of differentiation (Nelson et al., 2013). At E15.5, Sox9 was highly expressed at the tips of the epithelium where branching morphogenesis occurs and proacinar differentiation is initiated (Figure 1a), similar to that described previously (Chatzeli et al., 2017). In addition to expression in forming acini, Sox9 was also expressed in the intercalated ducts, adjacent to the acini, but was absent from the more proximally developing striated/granular and excretory ducts (Figure 1a′, a″) (Chatzeli et al., 2017). In the adult submandibular gland, three distinct populations of Sox9‐positive cells were observed (Chatzeli et al., 2017). A population with high levels of expression of Sox9 was located in the intercalated ducts (Figure 1b,d). These cells were negative for Mist1, a marker for acini cells in a variety of exocrine organs (Aure et al., 2015; Lemercier et al., 1997) (Figure 1b,c). In contrast to the high level of expression in developing acini (Figure1a–a″), adult acini expressed low levels of Sox9 overlapping with Mist 1 (Figure 1b,d). As in the embryo, the more proximally developing ducts (striated/granular, excretory) were devoid of Sox9 expression (Mist1 negative with large lumens) (Figure 1b,d). No obvious overlap was observed between the Sox9‐positive cells and myoepithelial cells, as labelled with α‐SMA (alpha smooth muscle actin) (Figure S1a,b). A similar expression pattern for Sox9 in adult glands has recently (been published using immunofluorescence and *Sox9creLacZ* mice, supporting our findings (Tanaka et al., 2019).

### 3.2 Fgf10 expression shifts from mesenchymal in embryonic glands to mainly epithelial in adult salivary glands

**FIGURE 2:**
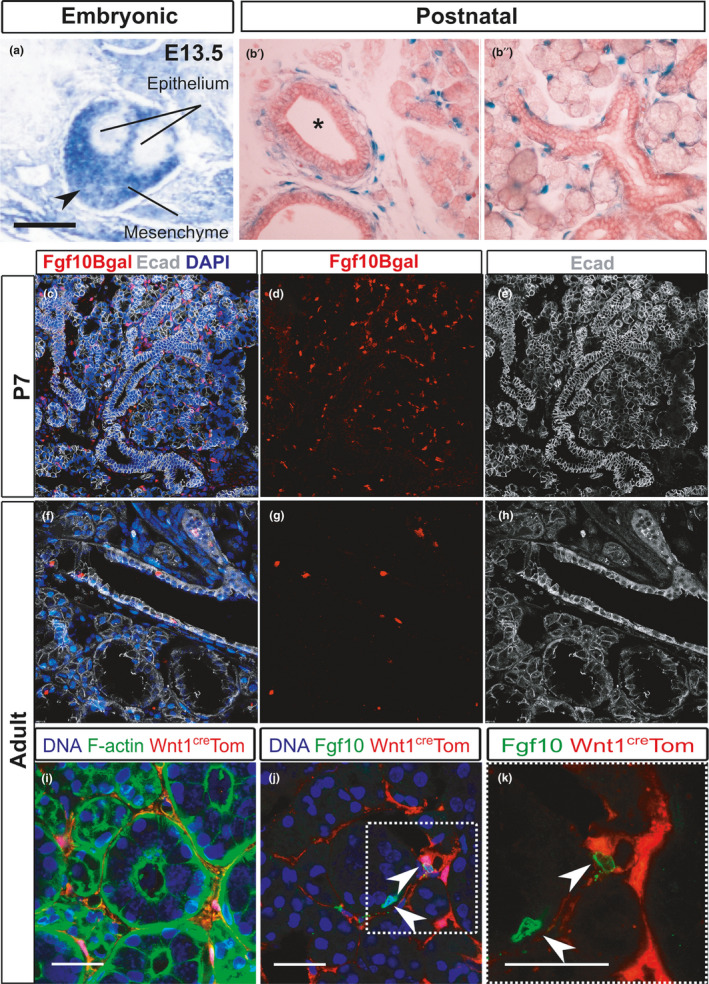
Fgf10 expression in the embryonic and adult submandibular glands. (a) *In situ* hybridisation for Fgf10 in the embryonic submandibular gland at E13.5. Arrowhead points to expression in the gland capsule. (b′–b″) LacZ staining (blue) of postnatal *Fgf10^LacZ^* mice 7 days after birth when the glands are still growing. Gland counterstained with eosin. (b′) Main excretory duct (asterisk) surrounded by positive cells. (b″) Forming striated ducts and acini are surrounded by positive cells in the adjacent mesenchyme. (c–e) Postnatal day 7. Immunofluorescence against B‐galactosidase (red), E‐cadherin (white), DAPI (blue). (c) Combined image showing Fgf10 cells in the mesenchyme. (d) Fgf10‐positive cells. (e) Gland morphology shown using E‐cadherin to outline the epithelium. (f–h) Adult male submandibular gland. Immunofluorescence against Bgal (red), Ecadherin (white) and DAPI (blue). (f) Combined image showing Fgf10‐positive cells in the gland epithelial ducts. (g) Fgf10‐positive cells. (h) Gland morphology shown using E‐cadherin to outline the epithelium. (i–k) Submandibular glands from adult female *Wnt1^cre^*;*R26^Tom^* mice. (i) Immunofluorescence for RFP (red) and F‐actin (green) to outline the gland structure. DNA is shown in blue (DAPI). Red cells are, as expected, located in the gland mesenchyme which is restricted in the adult. (j) Immunofluorescence against Fgf10 protein (green). Arrowheads highlight that Fgf10‐positive cells (green) do not overlap with the neural crest‐derived cells (red). Dotted box in j shown in (k). (k) Nonoverlap between *Wnt1creTom* and Fgf10. Scale bars i‐k: 25 µm

Having compared the pattern of Sox9 expression in embryonic and adult salivary glands we turned to investigate Fgf10, since Fgf10 and Sox9 are closely interlinked during development (Chatzeli et al., 2017). The mRNA of *Fgf10* in the embryonic glands was detected by *in situ* hybridisation (Figure 2a). The expression in the adult glands was detected both at the gene expression level using the reporter *Fgf10* line *Fgf10^nlacZ^* ^/+^ , where LacZ is under the control of *Fgf10* regulatory sequences without disrupting Fgf10 expression (Figure 2b–h) (Kelly et al., 2001), and at the protein level by immunofluorescence (Figure 2j,k). The *Fgf10^LacZ^* line, due to the higher half‐life of the β‐galactosidase as compared to *Fgf10* mRNA, marks both the *Fgf10* expressing cells and transiently their direct progeny that might no longer express *Fgf10* (Hajihosseini et al., 2008; Kelly et al., 2001). However, due to the slow turnover of the salivary gland tissue, it is highly likely that most or all of the X‐gal stained cells are the *Fgf10*‐expressing cells. As described previously, *Fgf10* was expressed throughout the neural crest‐derived mesenchyme of the embryonic glands (Figure 2a) (Jaskoll et al., 2005; Teshima et al., 2016; Wells et al., 2013). At postnatal stages when the glands are still developing, strong expression of Fgf10 was detected in the mesenchyme around the multi‐layered excretory duct (Figure 2b′), and in mesenchymal cells closely surrounding the acini (Figure 2b″, c, d, e). However, in the adult salivary glands, contrary to the widespread mesenchymal expression in the embryo, Fgf10 was expressed at much lower levels in the adult gland (Figure 2f–h). The majority of Fgf10‐positive cells were found in ductal epithelium (Figure 2f–h). As with Sox9, we saw no obvious overlap between expression of Fgf10 and myoepithelial cells (Figure S1b). Male submandibular glands (Figure 2f–h) showed higher numbers of Fgf10‐positive duct cells compared to female glands (Figure 6b,c), indicating that expression was mainly in the granular ducts, a specialised striated duct, which, due to sexual dimorphism, are more prominent in adult males (Gresik, 1975).

To investigate the origin of the cells expressed within the epithelium, we used the *Wnt1^cre^*;*R26^Tom^* mouse to label the progeny of neural crest cells, which in submandibular glands include the mesenchyme and the parasympathetic nerves (Figure 2i) (Ferreira & Hoffman, 2013; Jaskoll et al., 2002). No co‐localisation was observed between red fluorescence protein (RFP) and Fgf10, highlighting that adult Fgf10‐positive cells are not neural crest derived, and therefore a distinct lineage from the Fgf10‐expressing embryonic population (Figure 2j,k).

In the embryo, Fgf10‐expressing cells in the mesenchyme are located close to Sox9‐expressing cells in the distal gland epithelium (Chatzeli et al., 2017). In the adult gland, a few epithelial cells showing expression of both genes but the majority of Sox9 cells were not associated with Fgf10 cells, and visa versa (Figure S2). The relationship between these genes in the adult is therefore very different from the embryo.

### 3.3 The percentage of Sox9‐positive proliferating cells increases during regeneration

**FIGURE 3:**
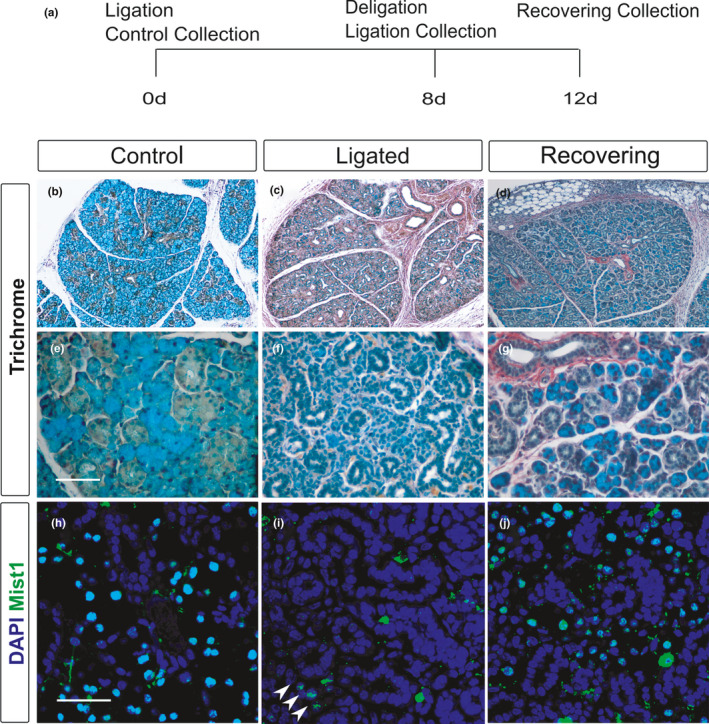
Degeneration of acinar cells after ligation and regeneration after deligation. (a) Experimental strategy to study submandibular gland regeneration after duct ligation. (b–g) Trichrome staining of an unoperated (b, e), ligated (c, f) and regenerating (d, g) submandibular gland. All mice were female 6–10 weeks old. (h–j) Immunofluorescence for the acinar cell marker Mist1 (green: nuclear stain) in an unoperated (h), ligated (i) and regenerating (j) submandibular gland. DNA is shown in blue (DAPI). Arrowheads in (i) indicate a few resistant acinar cells after ligation. Scale bars (e) and (h): 50 μm. Same scale in f,g,i,j

**FIGURE 4:**
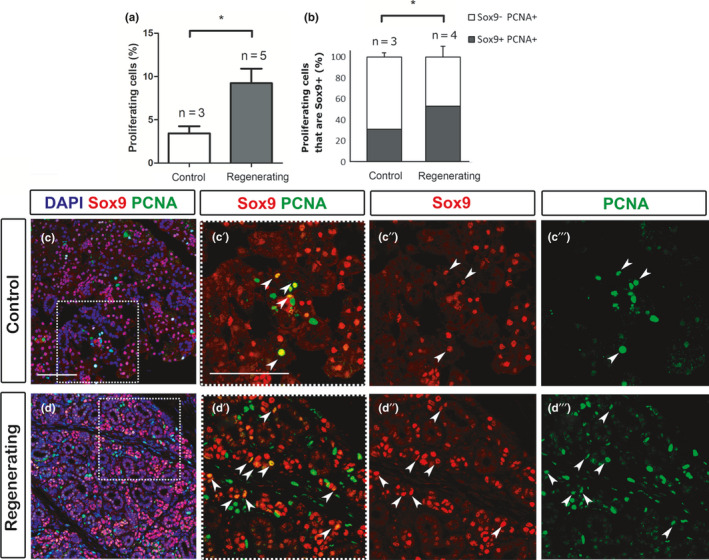
Sox9‐positive cells are highly proliferative in the regenerating submandibular gland. (a) Percentage of proliferating cells PCNA^+^/DAPI in the gland. (b) Percentage of proliferating cells that are Sox9‐positive (Sox9^+^PCNA^+^) and (Sox9^−^PCNA^+^) in the control unoperated and regenerating submandibular gland. Experiment follows the schedule shown in Figure 3a) with the ligation removed after 8 days and the animal culled 4 days later. *N* = number of mice. *p < 0.05. Error bars are s.e.m. (c, d) Immunofluorescence of Sox9 (pink) and PCNA (green). DNA is shown in blue (DAPI). All Sox9 expression is nuclear. (c–c′′′) Control unoperated. (d–d′′′) Regenerating submandibular gland. Dotted boxes in (c, d) indicate the magnified areas in c′, d′ respectively. (c′, c″, d′, d″) Sox9 (red) and (c′, c′′′, d′, d′′′) PCNA (green). Arrowheads indicate the Sox9^+^PCNA^+^ cells (yellow in c′,d′). All mice were female 6–10 weeks old. Scale bars in (c): 100 μm. Same scale in d. Scale bar in c′: 100 μm. Same scale in c″, c′′′, d′, d″, d′′′

Having established the expression of Sox9 and Fgf10 in the adult, we then turned to investigate how their expression changed during regeneration. For this, we used the salivary gland duct ligation model (Bozorgi et al., 2014; Cotroneo et al., 2008; Takahashi et al., 2004). Salivary gland ligation was performed using female mice for 8 days (Borzogi et al., 2014; Takahashi et al., 2004) and gland regeneration was followed for 4 days after the removal of the ligation (Figure 3a). This scheme allows for an investigation of the early response of tissue to injury (Borzogi et al., 2014; Cotroneo et al., 2008). Regeneration was examined by observing submandibular morphology using histological trichrome staining (Figure 3b–g) and by detecting the acinar cells based on their specific expression of Mist1 (Figure 3h–j). As previously described, atrophy was induced after 8 days of ligation (Figure 3b,c) (Cotroneo et al., 2008; Takahashi et al., 2004) with the glands losing most of their acinar cells (turquoise stain), and were mainly occupied by dilated ducts (Figure 3b,c) (*N* = 3). In agreement with other observations, a few resistant acinar cells were still present as detected by the expression of the acinar‐specific marker Mist1 (Figure 3i arrowheads) (Aure et al., 2015; Cotroneo et al., 2008; Takahashi et al., 2004). These resistant acinar cells are thought to be the ones that can rescue the acini after deligation (Weng et al., 2018). When salivary glands were left to regenerate for 4 days after the clip was removed, more acinar cells were observed as shown by Alcian blue positive cells after trichrome staining (*N* = 5) (Figure 3d,g) and Mist1 immunofluorescence (Figure 3j), suggesting that acinar cells have been produced by a process of regeneration. As expected, proliferation levels, as observed by PCNA staining, significantly increased in the regenerating (deligated) salivary glands compared to age‐ and sex‐matched control glands (Figure 4 a, c′′′, d′′′), agreeing with previously described research using this model (Takahashi et al., 2004). In control adult female salivary glands, only 3.5% of the total number of cells underwent proliferation (Figure 4a) (*N *= 3) compared to 9% during gland regeneration (*N* = 5; Figure 4a). Having confirmed that atrophy and regeneration were occurring in our model, the expression of Sox9 was investigated. In the kidney, the Sox9‐expressing population is the main source of proliferating cells during the regenerative process (Kang et al., 2016). Similarly, overexpression of Sox9 in a duct cell line was shown to increase proliferation (Tanaka et al., 2019). Therefore, the contribution of Sox9‐positive cells to proliferation during salivary gland regeneration was investigated (Figure 4c,d). At both stages there are many more Sox9 cells than proliferating cells (Figure 4c–c′′′, d–d′′′), highlighting that not all Sox9 cells are activity proliferating. In the control glands, 31% of all PCNA‐positive cells were Sox9 positive (*N* = 3), while in the deligated regenerating gland 53% of all PCNA‐positive cells were Sox9 positive (*N* = 4) (Figure 4b). Therefore, the percentage of Sox9‐positive proliferating cells almost doubled during regeneration constituting half of the total proliferating population.

### 3.4 Ectopic expression of Sox9 in striated/granular ducts during regeneration

**FIGURE 5:**
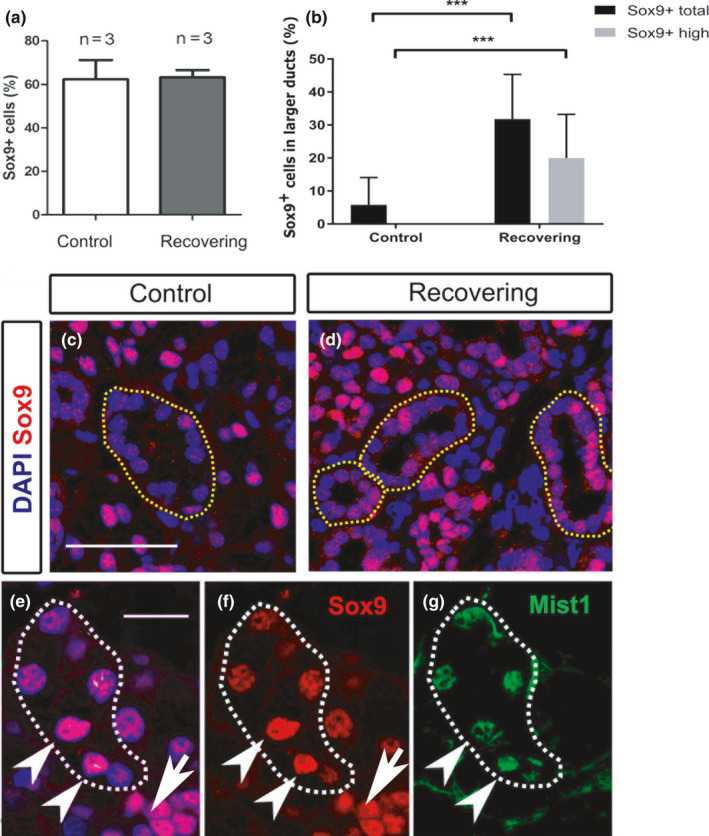
Ectopic expression of Sox9 in striated/granular duct cells. (a) Percentage of Sox9‐positive cells (Sox9^+^/DAPI) in the control‐unoperated and regenerating submandibular gland. *N* = number of mice. (b) Percentage of total Sox9‐positive cells (low and high expression) referred to as Sox9^+^ total (Sox9^+^ total/DAPI) and percentage of highly expressing Sox9‐positive cells, referred to as Sox9^+^ high (Sox9^+^ high/DAPI) found in the striated/granular ducts of control and regenerating submandibular glands. ***p < 0.001. Error bars are s.e.m. (c, d) Immunofluorescence of Sox9 (pink) in the control (c), and regenerating (d) submandibular gland. Dotted yellow lines delineate the striated/granular ducts. (e–g) Immunofluorescence for Sox9 (red) and Mist1 (green) in the regenerating submandibular gland acini. Dotted white line in (e‐g) delineates a Mist1‐positive acinus. (e) Sox9 (red) overlaps with DAPI (Blue) in the nucleus. (f) Sox9. (g) Mist1. Arrowheads (e–g) point to acinar cells (Mist1 positive) with strong Sox9 expression. Arrows point to intercalated ducts with strong Sox9 expression but no Mist1. All mice were female 6–10 weeks old. Scale bar (c): 50 μm, same scale in (d). (e) 25 μm, same scale in (f, g)

Salivary gland development is characterised by elevated Sox9 expression in the highly proliferative distal epithelium (Chatzeli et al., 2017). To investigate whether regeneration was associated with changes to Sox9 expression, the number of Sox9‐positive cells was quantified in control and regenerating salivary glands as a proportion of the total number of cells in an equivalent region (Figure 5a) (*N* = 3 for each condition). Interestingly, the number of Sox9‐positive cells remained constant (Figure 5a), despite the greater number of proliferating Sox9 cells in the regenerating glands (Figure 4b). The increase in proliferating Sox9 cells could be offset by the reduced number of acinar cells in the regenerating glands as compared with the unoperated mice (Figure 3b,d). In addition, in the regenerating glands non‐Sox9‐positive cells might turn on Sox9 expression during regeneration. To look at this in more detail, we analysed the distribution of Sox‐9‐positive cells in the operated glands versus the controls (Figure 5c–g). Strikingly, although only a very few weakly Sox9‐positive cells were evident in striated/granular ducts in the control glands (Figures 1d and 5c), large numbers of positive cells were evident in these ducts in deligated glands (Figure 5d). The proportion of Sox9‐positive cells found in striated/granular ducts was quantified for each condition (Sox9^+^ total), along with the proportion of highly expressing Sox9‐positive cells (Sox9^+^ high) (Figure 5b). In control mice, less than 6% of the striated/granular duct cells were Sox9 positive. Of the few cells that were Sox9 positive all expressed Sox9 at low levels. In contrast, in the regenerating condition, this number rose to 32% with 20% having high levels of Sox9 expression. Upregulation of Sox9 in these larger ducts therefore appears to be an important feature in injury and regeneration.

In addition to the change in expression in the large ducts, a change in the regenerating acini was also noted (Figure 5e–g). In control glands, Sox9 was expressed at lower levels in the acini when compared to the neighbouring intercalated ducts (Figure 1d). In the regenerating glands after deligation, two patterns of expression were observed in the acini. In some cases, the Mist1‐positive regenerating acini expressed high levels Sox9, reminiscent of levels observed during development, while in other cases the levels of Sox9 were lower in these Mist1‐positive cells (Figure 5e–g), more similar to the pattern in control adults (see Figure 1). These variations may represent different stages of acinar regeneration. The neighbouring intercalated ducts also showed high levels of expression of Sox9 (Figure 5e–g).

### 3.5 Fgf10‐positive cells increase during regeneration but maintain their expression in the gland epithelium

**FIGURE 6:**
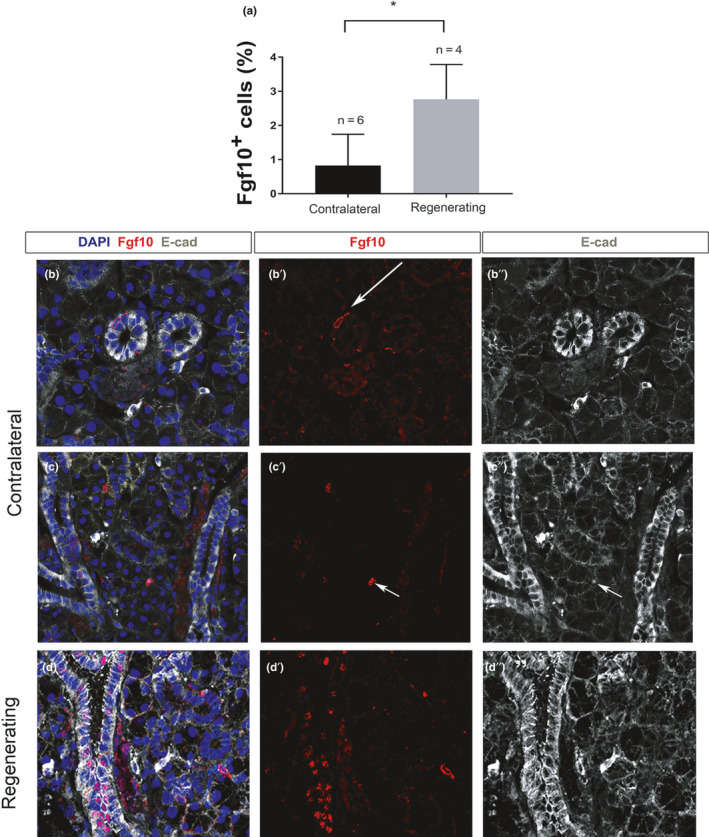
Fgf10‐positive cells increase in the ductal epithelium during regeneration. (a) Percentage of Fgf10‐positive cells in control and regenerating submandibular glands (% of total cells). *p < 0.01. (b, c) Immunofluorescence in contralateral (b, b′, b″ and c, c′, c″), and regenerating (d, d′, d″) female submandibular glands. (b, b″, c, c″, d, d″) Epithelium outlined with Ecadherin (white). DNA is shown in blue (DAPI). (b, b′, c, c′, d, d′) Fgf10 immuno (red). (b, b′, b″) Only a few Fgf10‐positive cells are identified in the duct epithelium (arrow). (c, c′ c″) Sparce Fgf10 cells overlap with E‐cadherin (arrow) indicating expression in the epithelium. (d, d′, d″) Multiple Fgf10‐positive cells are identified during regeneration, the majority located in the striated/granular ducts. All mice were female 6–10 weeks old

Having analysed the pattern of Sox9 expression in the homeostatic and regenerating salivary gland, we then moved on to investigate Fgf10 expression. Fgf10 is an important mitogen of epithelial progenitors during salivary gland embryonic development (Jaskoll et al., 2005; Lombaert et al., 2013; Steinberg et al., 2005), and therefore it might be predicted to be elevated during regeneration. We quantified the number of Fgf10‐positive cells in lobes from contralateral and regenerating female salivary glands (Figure 6a–d). The Fgf10‐positive cells were significantly increased from 1% during homeostasis (*N* = 6) to 3% of the total number of cells during regeneration (*N* = 4), indicating a response to injury (Figure 6a). Fgf10‐positive cells were largely detected in the striated/granular ducts of regenerating glands (Figure 6d–d″). Importantly, an obvious upregulation of Fgf10 in the mesenchyme surrounding the regenerating acini was not observed, as would have been predicted if regeneration was closely mimicking development.

## 4 DISCUSSION

Salivary glands are important organs for dental health and well‐being. Currently, around 27% of the elderly, and head and neck cancer patients (10% of malignant tumours) undergoing irradiation treatment suffer from salivary gland dysfunction, which could be treated by organ regeneration (Gupta et al., 2006; Vissink et al., 2003). Although salivary gland regeneration has been proposed to mimic embryonic development (Cotroneo et al., 2010; Patel & Hoffman, 2014), few studies have investigated the extent in which the developmental programme correlates with regeneration. Here, we compared the expression of two highly interlinked and essential genes for salivary gland development, *Sox9* and *Fgf10* during salivary gland development, homeostasis and regeneration. We found that although there is an elevated appearance of Fgf10 and Sox9‐positive cells during regeneration, their function during regeneration may be different from that during organogenesis.

### 4.1 During regeneration Sox9 is turned on in striated/granular ducts, a location with low Sox9 levels during development and homeostasis

If regeneration recapitulates embryonic development, it could be speculated that genes expressed during development would be expressed in similar locations during regeneration. During branching morphogenesis in the embryo, Sox9 was highly expressed at the tips of the developing epithelium and absent from the proximal structure that will form the main duct network (Chatzeli et al., 2017). Similar to the developing gland, Sox9 was expressed in the most distal epithelium of adult submandibular glands, the acini and intercalated ducts, which are the progeny of the embryonic distal epithelium (Matsumoto et al., 2016) and was mainly absent from the striated/granular and excretory ducts. Sox9 has recently been suggested to regulate a putative stem cell population of CD133 expressing cells in the adult gland (Tanaka et al., 2019). Contrary to homeostasis, in regenerating salivary gland, Sox9 was upregulated in many epithelial cells housed within the striated ducts. This novel expression domain suggests a new function for Sox9 in ductal cells during regeneration that is distinct from its role during development and homeostasis. This upregulation might be linked to the activation of a suggested population of adult progenitors within the ducts that contributes to the production of distally located ductal cells (Pringle et al., 2013).

In contrast to the novel expression in the ducts, high levels of expression of Sox9 were also observed in a few acinar cells in the regenerating glands, suggesting that Sox9 upregulation is involved in formation of new acini during repair, potentially recapitulating its role in acini development during embryogenesis. High and low levels of Sox9 expression have previously been described in the developing pancreas, with the low state correlating with lower levels of Fgfr2 expression (Seymour et al., 2012).

### 4.2 Mesenchymal Fgf10 expression does not recur in the regenerating salivary gland

Salivary gland embryonic development is characterised by high levels of Fgf10 expression in the neural crest‐derived mesenchyme of the gland during initiation and branching morphogenesis (Jaskoll et al., 2005; Teshima et al., 2016; Wells et al., 2013). This reliance on mesenchymal expression of Fgf10 is highlighted by the fact that the conditional *Fgf10* knockout in the neural crest (*Wnt1‐cre* driven) has the same early arrest of the salivary gland placode as the full *Fgf10* knockout (Teshima et al., 2016). However, in the lobes of the adult submandibular gland, Fgf10 expression was limited and confined to the epithelium. Some Fgf10‐positive cells were located in the interlobular connective tissue and capsule, which has a mesenchymal origin, however, these cells were very rare. During regeneration, the number of Fgf10 expressing cells was upregulated from 1% to 4% of the total gland, but still represented a small number of cells overall. The upregulation was most obvious in the striated/granular ducts but no new expression was observed in the mesenchyme around the regenerating acini, as might be predicted if regeneration directly recapitulated development. The role of Fgf10 in regeneration therefore appears distinct from development.

### 4.3 Fgf10 and Sox9 are both upregulated in striated ducts during regeneration

During salivary gland development, Sox9 expression is regulated by Fgf10 (Chatzeli et al., 2017), this relationship being conserved during the development of a number of other branching organs (Abler et al., 2009; Chang et al., 2013; Chen et al., 2014; Seymour et al., 2012). In the developing gland, their spatial and temporal expression is tightly linked from the initiation stage of salivary gland development, with Fgf10 expressed in the mesenchyme and Sox9 in the epithelium (Chatzeli et al., 2017; Jaskoll et al., 2005; Wells et al., 2013). As branching morphogenesis proceeds, Sox9 is highly expressed at the tips of the epithelium that will generate the acinar cells and smaller ducts while it is reduced at cells that form the excretory duct (Chatzeli et al., 2017; Matsumoto et al., 2016), with Fgf10 expressed in the mesenchyme that surrounds these distal structures (Figure 2). Interestingly, given their close association in the developing gland, the expression pattern of Fgf10 and Sox9 in the adult submandibular gland was largely distinct, with Fgf10 concentrated in more proximal epithelial, while Sox9 was largely excluded from this region (Figure S2). This suggests that the regulation of Sox9 by Fgf10 reported in the embryonic salivary gland (Chatzeli et al., 2017) is not maintained in the adult gland.

**FIGURE 7:**
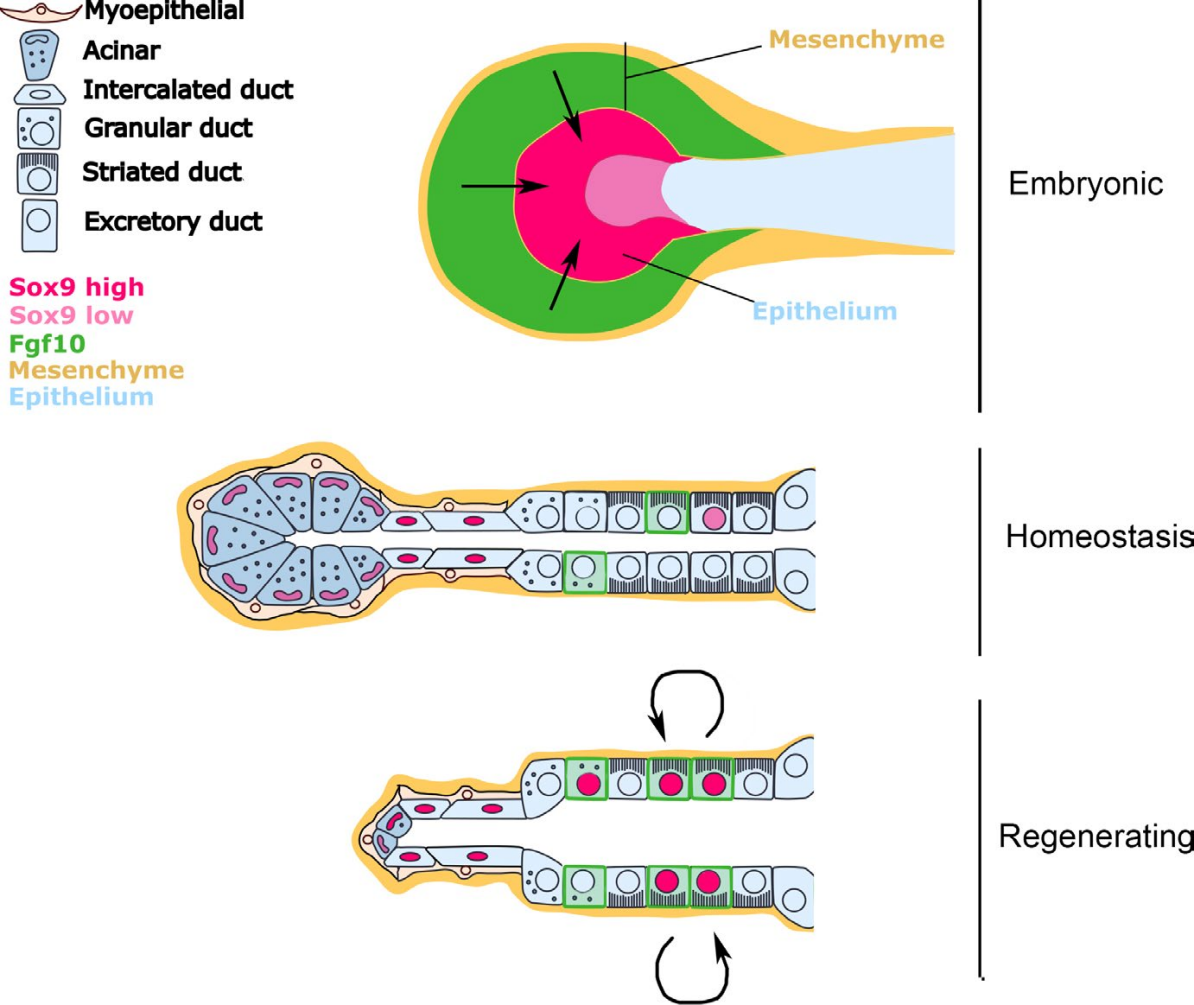
Schematic of the expression pattern of Fgf10 and Sox9 during salivary gland organogenesis, homeostasis and repair. Fgf10 is shown in green while Sox9 is shown in pink. During development, mesenchymal Fgf10 signals to epithelial Sox9 and directs expression in the distal region of the gland. During homeostasis, the expression of Fgf10 shifts to the ductal epithelium, while Sox9 remains expressed in the distal part of the glands (acini and intercalated ducts). During regeneration Sox9 and Fgf10 are upregulated in the ducts and may interact. Black arrows indicate a regulatory mechanism between Fgf10 and Sox9. This has been confirmed during development and needs to be tested during regeneration

During regeneration, however, both Fgf10 and Sox9 were upregulated in the granular/striated ducts, suggesting a re‐awakening of this regulatory relationship during regeneration. The changing pattern of expression and potential regulatory relationships of Fgf10 and Sox9 during regeneration is illustrated in Figure 7. A number of duct progenitor cells markers have previously been identified (Keratin 5, Keratin 14, Axin 2, c‐Kit, Ascl 3), which appear to label distinct populations within different ducts (Bullard et al., 2008; Kwak et al., 2016; May et al., 2018; Rugel‐Stahl et al., 2012; Weng et al., 2018). Using the duct ligation model, Keratin 5 and Axin 2 duct progenitor cells have been shown to be able to regenerate duct cells, but not cells of the acinar lineage (Weng et al., 2018). It would therefore be interesting to investigate the relationship of Sox9 and Fgf10 expressing cells to these other markers of duct stem/progenitors cells, and to test their potential using lineage labelling studies.

The experiments performed here rely on the duct ligation model to trigger gland atrophy and regeneration. An alternative model used to study regeneration is the irradiation model, which more closely recapitulates the injury observed after radiation therapy in head and neck cancer patients. The two models have different degrees of regeneration in mouse models, with irradiation leading to a more significant, long‐term, damage to the gland and less robust regenerative capacity (Weng et al., 2018). The irradiated gland, however, does undergo some regeneration, with Sox2 cells replenishing the acinar compartment of the sublingual gland (Emmersen et al., 2018). The two methods share some similarities, for example, p63 is upregulated after both techniques (Ikai et al., 2020). It would therefore be interesting to study whether the changes in Fgf10 and Sox9 expression observed here after gland ligation also occur after irradiation.

Overall our data indicate that Sox9 and Fgf10 regulation during regeneration in adult glands does not directly mimic development. We see no widespread upregulation of Fgf10 in regenerating gland mesenchyme, while we observe the induction of Sox9 and Fgf10 in tissues that do not express these genes in the embryo. The regulatory relationship between these two factors established in the embryo also does not appear to be conserved during homeostasis but may be re‐established during regeneration (Figure 7). It seems, therefore, that regeneration uses similar genes to development but does not directly recapitulate the embryonic programme, perhaps due to differences in the cell types generated and the microenvironment of the tissue. Learning about development can therefore shed light on the genes that are likely to be involved during regeneration but regeneration should not be presumed to always follow similar rules.

## CONFLICT OF INTEREST

Abigail Tucker is a council member of the Anatomical Society.

## 5 FUNDING

LC was funded by the Anatomical Society from a PhD studentship awarded to AT and GP.

## AUTHOR CONTRIBUTIONS

Experiments were designed by A.S.T, G.B.P. and L.C. Duct ligation experiments were performed by L.C. and G.B.P. Gland analysis was performed by L.C., M.G., T.H. and A.S.T. LacZ samples were provided by M.K.H. Figures were designed by L.C., M.G. and T.H. Manuscript was written by L.C. and A.S.T. All authors read and critically revised the manuscript.

## Supporting information

Figure S1

Figure S2

## Data Availability

All mouse lines used in this research are available for sharing. All reagents are commercially available.
