# Supplementary figures and images for: Comparing development and regeneration in the submandibular gland highlights distinct mechanisms
Source: J Anat. 2021 Jan 16;238(6):1371–85. doi: 10.1111/joa.13387 (PMC8128775; doi:10.1111/joa.13387)

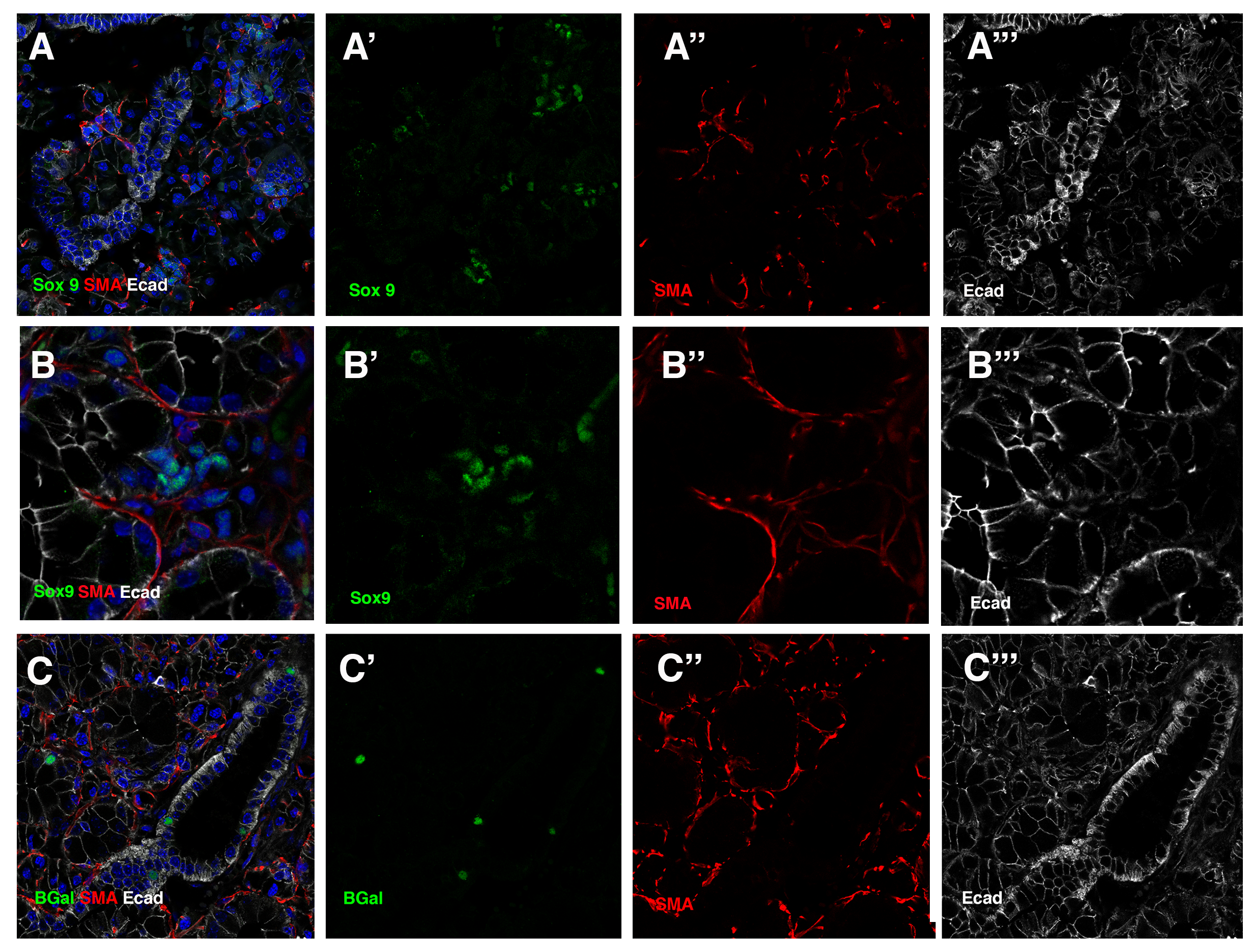

Supplement: Supplementary file 1 — Figure S1 [file JOA-238-1371-s002.tif]

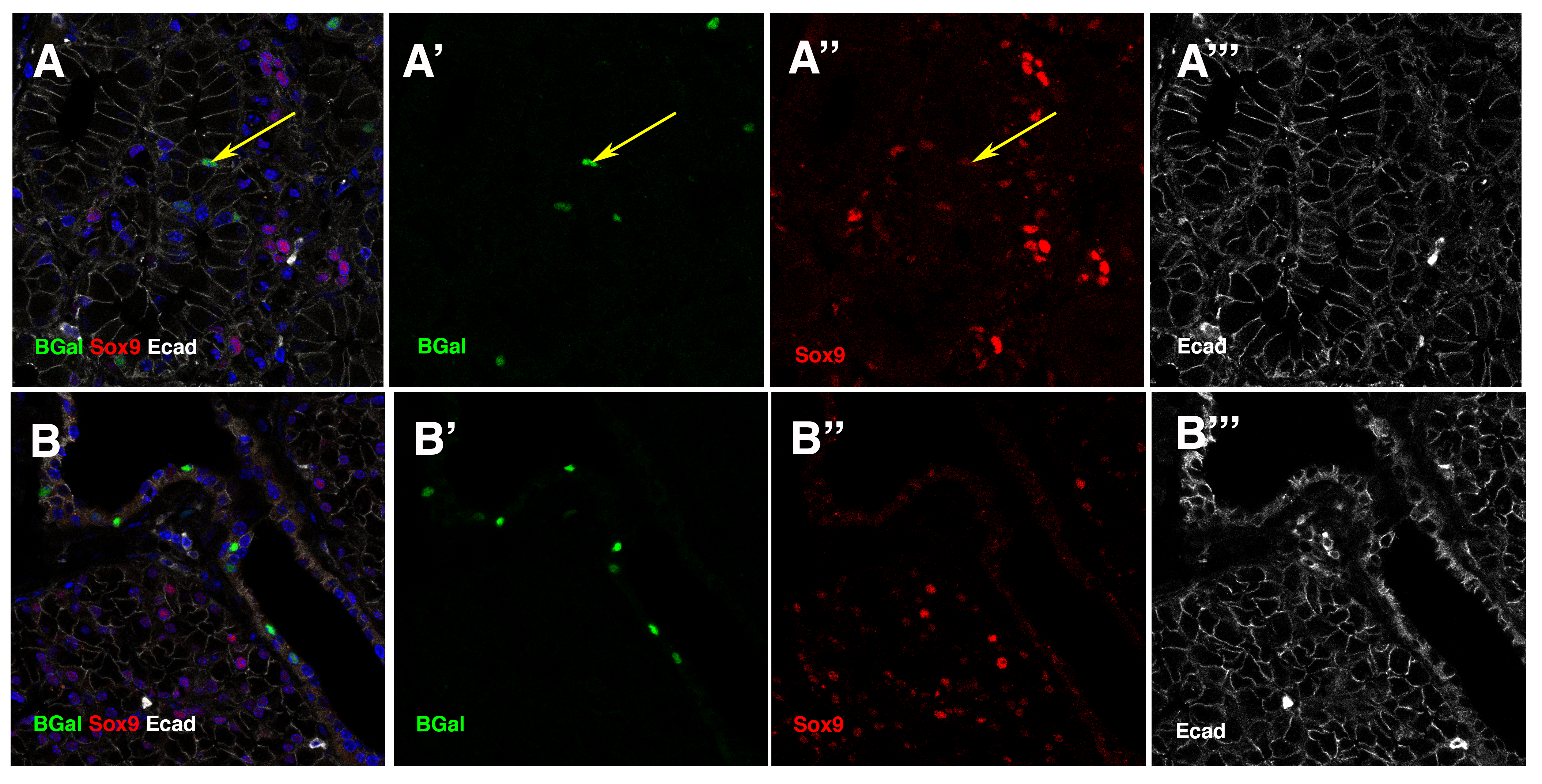

Supplement: Supplementary file 2 — Figure S2 [file JOA-238-1371-s001.tif]
